# Evaluation of an autonomous smart system for optimal management of fertigation with variable sources of irrigation water

**DOI:** 10.3389/fpls.2023.1149956

**Published:** 2023-04-12

**Authors:** Alberto Imbernón-Mulero, José F. Maestre-Valero, Victoriano Martínez-Alvarez, Francisco J. García-García, Francisco J. Jódar-Conesa, Belén Gallego-Elvira

**Affiliations:** ^1^ Deparment of Agricultural Engineering, Technical University of Cartagena, Cartagena, Spain; ^2^ Technical Direction, Nutricontrol S.L., Calle Bucarest, Cartagena, Spain; ^3^ Agrícola Conesa Martín S.L., Torre Pacheco, Spain

**Keywords:** non-conventional irrigation water, precise fertigation, high-tech irrigation head, nutritional adjustment, interoperability

## Abstract

Modern irrigation technologies and tools can help boost fertigation efficiency and sustainability, particularly when using irrigation water of varying quality. In this study, a high-tech irrigation head using a new fertigation optimization tool called NutriBalance, which is designed to manage feed waters of different qualities, has been evaluated from technical and economic perspectives. NutriBalance computes the optimal fertigation dose based on specific data about the equipment, the crop, the irrigation water, and the fertilizers available, in order to enable autonomous and accurate water and fertilizer supply. The system was trialed in a grapefruit orchard irrigated with fresh and desalinated water for several values of crop nutritional requirements and considering different fertilizer price scenarios. The results showed the good interoperability between the tool and the irrigation head and the nearly flawless ability (error below 7% for most ions) of the system to provide the prescribed fertigation with different combinations of irrigation water. Fertilizer savings of up to 40% were achieved, which, for the lifespan of the equipment, were estimated to correspond to around 500 EUR/ha/year. The results of this study can encourage the adoption of novel technologies and tools by farmers.

## 1 Introduction

The decrease in freshwater resources across the world (Jones et al., 2019; Redondo-Orts and López-Ortiz, 2020) has led to unprecedented pressure on irrigated agriculture as well as threats to food security (Jiang et al., 2022). In this context, the use of non-conventional water resources has become a promising solution for irrigation in water-starved regions (Pedrero et al., 2015; Martínez-Alvarez et al., 2020; Redondo-Orts and López-Ortiz, 2020). Desalinated seawater (DSW) is being increasingly seen as a viable option to sustain agricultural needs in coastal areas, since it provides a steady water supply that overcomes climatological and hydrological constraints (March et al., 2014; Reca et al., 2018; Jones et al., 2019). In addition to DSW, water reused after purification and regeneration processes, *i.e.*, reclaimed water, is providing a supplementary supply to conventional sources (Martínez-Alvarez et al., 2019; Mainardis et al., 2022).

Nevertheless, the use of these sources adds a new layer of complexity to fertigation, owing to their particular characteristics. In particular, DSW has a low salinity, with low concentrations of several essential nutrients (Ca^2+^, Mg^2+^ and SO_4_^2–^) and high concentrations of others (B^3+^, Cl^-^ and Na^+^), which may imply phytotoxicity effects (Martínez-Alvarez et al., 2017). On the contrary, reclaimed water has higher salinity but can provide several nutrients, such as nitrates, phosphates, potassium, calcium and magnesium, which are scarce in other resources (Maestre-Valero et al., 2019). Therefore, the use of these resources in combination with conventional waters is recommended (Martínez-Alvarez et al., 2017; Reca et al., 2018).

Another sustainability concern of irrigated agriculture is the overuse of fertilizers, which implies agronomic, environmental and profitability hazards. The excessive supply of nutrients can harm crops and causes soil degradation and aquifer contamination (Solgi et al., 2018; Ashitha et al., 2021). Moreover, the price of fertilizers has soared in the last two years, driven by the scarcity of raw materials (IndexBox, 2022; MordorIntelligence, 2022; Chojnacka et al., 2023; Lahmiri, 2017). For instance, the price of simple inorganic fertilizers rose from 0.60 EUR in 2021 to 1.60 EUR in 2022 in Spain (IndexBox, 2022).

To address the current water and fertilizer crisis, it is essential that irrigators implement resource-efficient systems (Zhang and Guo, 2016; Fereidoon and Koch, 2018). Pressurized (drip and sprinkler) irrigation systems provide higher uniformity and lower loses than traditional (gravity) surface methods (Tarjuelo et al., 2015; Nikolaou et al., 2020). In particular, modern drip irrigation systems can be adapted to optimize fertigation in a wide array of climate and soil conditions (Suárez-Rey et al., 2020; Obaideen et al., 2022). Their efficiency can be maximized using high-tech irrigation heads and new generation (self-compensating, underground) drippers (García et al., 2020), soil and plant monitoring sensors (Shafi et al., 2019), irrigation time and volume control devices (Safdar-Munir et al., 2019), and wireless communication and data acquisitions systems (Elsayed et al., 2021).

However, the vast majority of drip irrigation systems remain to a certain extent “rudimentary”, due to the low adoption of technology and tools by farmers (Bondesan et al., 2023). Smart systems still need to become more accessible and user-friendly for farmers to boost their adoption (Li and Wang, 2021). The calculation of fertilizer dose and irrigation time is usually based on the farmer’s experience and available documentation (Barry-Stelljes, 2000; Mainardis et al., 2022). Recent studies show that farmers mostly use: (i) crop evapotranspiration estimation methods (FAO56─Penman Monteith; Allen et al., 1998), (ii) nutrient requirements recommendations from the literature (Mainardis et al., 2022), (iii) some soil and plant sensors (Gallardo et al., 2020), and (iv) pH and EC controllers in irrigation heads (Zaman et al., 2018). Monitoring often covers only basic parameters (Mourya and Lal-Mourya, 2015; Zaman et al., 2018), so episodes of nutritional deficit or excess are likely to occur, particularly when using variable quality irrigation water.

The integration of recent smart tools can help optimize fertigation processes (Seethalakshmi et al., 2021), and even adapt dosage in real time to irrigation waters of fluctuating composition (Avni et al., 2013; Li and Wang, 2021). Several fertigation tools can be found in the literature for managing the application of fertilizers in soil and soilless crops (review by Gallardo et al., 2020). Many such tools calculate the optimal combination of fertilizers to meet the nutritional needs (Moreira-Barradas et al., 2012; Pérez-Castro et al., 2017), while some of them can also provide the most cost-effective fertigation option (Bueno-Delgado et al., 2016), considering climate, soil conditions, type of crop and available resources and infrastructures. More recently, a few tools have been proposed for the management of waters of different quality. Reca et al., 2018 presented the program GARUM that computes the optimum mixture of desalinated and brackish water for greenhouse crop irrigation. Afterwards, Gallego-Elvira et al., 2021 developed the tool Irriblend-DSW, which computes the optimum blend of waters with different quality and price and the most profitable combination of commercial fertilizers. These tools were designed to curb fertigation costs when using DSW for irrigation, as well as to prevent over-fertilization and leaching of nutrients.

The principal aim of the present study was to implement a high-tech fertigation system and test its efficacy in accurately providing different concentrations of fertilizer under varying water quality conditions. The system included a novel optimization tool named NutriBalance, which has an algorithm that can autonomously manage fertigation with feed waters of different quality. Both the technical and the economic viability of the system have been assessed for the first time in a commercial grapefruit orchard. A citrus crop was chosen for this study for two reasons: (i) Spain is the leading producer of citrus in Europe and the Mediterranean region, and (ii) precise irrigation of citrus trees is particularly important, since phytotoxic effects (mostly due to boron excess) have been observed following the massive introduction of DSW for irrigation (Martínez-Alvarez et al., 2020).

## 2 Materials and methods

### 2.1 Study site

The experimental work was carried out between January 2021 and December 2022 at a commercial citrus farm of 3.18 ha located in Torre Pacheco, Spain (37°47’30” N; 1°03’85” W; 30 m above sea level). The study area has a semiarid climate with hot and dry summers and mild-temperature winters. Sporadic intense rains concentrate in autumn, and crops mostly depend on irrigation. For the evaluation of the experimental system, an area of 0.28 ha was delimited, in which there were 144 grapefruit trees (*Citrus x paradisi* var. Rio Red) of two and three years of age.

### 2.2 Description of research equipment

A high-tech irrigation head was deployed to enable autonomous and accurate fertilizer dosing by drip irrigation with the NutriBalance optimization program (described in section 2.3).

**Figure 1:**
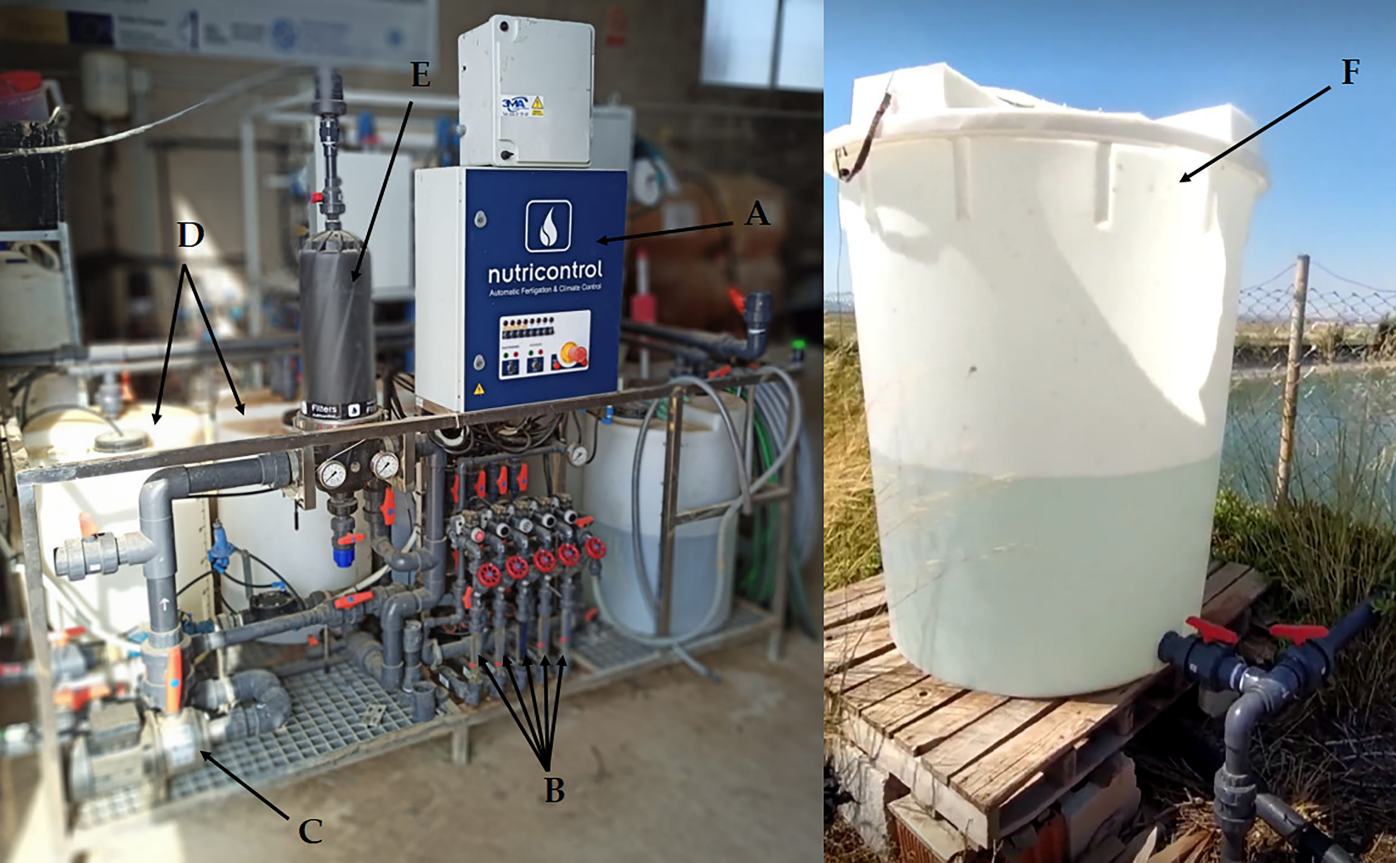
Experimental irrigation head. **(A)** Control panel; **(B)** fertilizer injectors; **(C)** feed pump; **(D)** fertilizer tanks; **(E)** ring filter; **(F)** sampling tank.

The technical specifications of the irrigation head are as follows. Its dimensions were 1.600 m (height) x 2.250 m (length) x 1.125 m (width), and its weight was around 200 kg. **Figure 1** shows the irrigation head, which consisted of four different subsystems:

(i) The hydraulic subsystem, which consisted of a 4.5-bar feed pump (Franklin Electric, Model EH 9/3, 1.1 kW) working at a flowrate of 1.2 m^3^/h, a collector with a pH meter (HT3 glass, BNC coax pH probe) and EC probes (EC BK 0.005 to 10 dS/m) and a 2” ring filter to retain particles up to 130 μm.
(ii) The fertilizer injection subsystem, which had five closed polyethylene (PE) fertilizer tanks (125 L), a single-phase air agitator (1.4 kW, 2500 rpm) to facilitate the dissolution of the fertilizers, five flow meters with a maximum flow rate of 1500 L/h (continuous fluid signal with 1000 pulses per liter) and five venturi injectors with 500 L/h meters, 4 mm flip solenoid valves, and manual and one-way valves.
(iii) The electrical subsystem, which included an electrical panel, protection devices and the control drivers of the hydraulic and fertilizer injection subsystem.

The automaton for fertigation management, which integrated the optimization software, the user interface and the communication system with the cloud server.

### 2.3 Description of fertigation program NutriBalance

The fertigation program NutriBalance was developed to enable the accurate supply of different concentrations of fertilizer under different water quality conditions. The farmers in the study area use DSW alone or combined with other resources, depending on availability. Therefore, the fertigation system should be ready to handle such fluctuations in input water composition.

**Figure 2:**
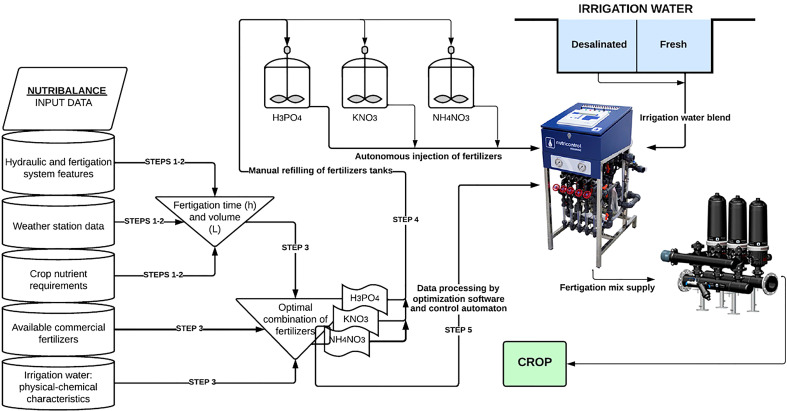
Outline and workflow of the experimental system.

NutriBalance was integrated into the high-tech irrigation head described in the previous section. **Figure 2** shows the outline and workflow of the experimental system (irrigation head plus NutriBalance). NutriBalance requires the following specific information, which must be provided by the user:

- Technical characteristics of the hydraulic and fertigation system, including the number of drippers per tree, the flow rate of the drippers and the volume of the fertilizer tanks.
- Weather station data and crop coefficient to calculate FAO Penman-Monteith ET_0_ (Allen et al., 1998).
- Crop nutrient requirements.
- Commercial fertilizers available at the field, indicating price and physical and chemical properties.
- A physical and chemical analysis of the irrigation water, indicating ion composition.
- User restrictions: maximum values of pH or EC in the nutrient solution.

With said information, NutriBalance computes the optimal fertilizer dose as follows:

1. Calculation of water requirements and irrigation time: The weekly water requirements of the crop are calculated as the crop evapotranspiration under standard conditions (ET_c_) following the crop coefficient method (Allen et al., 1998) minus rainfall (R). The gross water requirement per plant (*N*_G_, L/plant/day) is calculated accounting for the uniformity coefficient (*UC*, %) and the transpiration relation (*T*_r_, dimensionless) as follows:


(1) $$N_{G}=\frac{\sum^{}ET_{c}-R}{\frac{UC}{T_{r}\cdot 100}\cdot }\cdot \frac{1}{10}$$

Then the minimum irrigation time, *T*_a_ (h), is calculated using the following equation:


(2) $$T_{a}=\frac{N_{G}}{\frac{n_{drippers}}{tree}\cdot q_{dripper}}$$

where, *n*_drippers_ is the number of drippers used per tree and *q*_dripper_ is the flow rate of the drippers (L/h).

2. Calculation of fertigation solution: based on the computed water requirements and the nutrient needs indicated by the user, the fertigation volume (*V*_irrigation_, water + fertilizer) needed per week for a target crop area is calculated as follows:


(3) $$V_{irrigation}\left(L\right)=\frac{\frac{n{{}^{\circ}}_{drippers}}{tree}\cdot q_{dripper}\cdot \frac{trees}{hectare}\cdot \frac{IH}{week}}{1000}\cdot 1000$$

where, *IH* are the hours of irrigation (h).

3. Selection of the best combination of commercial fertilizers considering the composition of the water blend. The optimization algorithm described in Martínez-Alvarez et al., 2020 is used, which determines the type and amount of commercial fertilizer that can provide the nutrients needs at minimum cost. The algorithm uses a constrained minimization with sequential least squares programming to minimize the cost function considering the EC and pH constraints provided by the user.

4. Specific volume of water and fertilizer in each fertilizer tank. The final concentration of each fertilizer in the tank is calculated as follows:


(4) $$F_{max}\left(L\right)=\frac{V_{tank}}{1+\left(\frac{\rho_{f}}{LS}\right)}$$

where, *F*_max_ is the maximum amount of fertilizer per tank (L), *V*_tank_ is the volume of the tank (L), *ρ_f_* is the density of the fertilizer (g/L) and *LS* is the minimum solubility of the fertilizer (g/L).


(5) $$W_{d}\left(L\right)=\left(\frac{1}{LS}\right)\cdot F_{max}\cdot \rho$$

where, *W*_d_ is the water required to dilute the fertilizer.


(6) $$C_{f}\left(g/L\right)=\frac{\frac{F_{max}\cdot \rho_{f}}{MW_{f}}\cdot MW_{ion}\cdot V\cdot 1000}{W_{d}}$$

where, *C_f_* is the final concentration of each fertilizer in the tank, *MW*_f_ is the molecular weight of the fertilizer, *MW*_ion_ is the molecular weight of a specific ion and *V* is the valence of the ion.

5. Finally, the calculated water and fertilizer volumes are sent to the control automaton.

It is important to note that the fertilizer tank must always have the same fertilizer concentration (mg/L) in order to perform adequate adjustment of the volume of fertilizer. It is therefore a fixed parameter, which the farmer must keep constant when refilling the fertilizer tanks.

The link and images of the online demonstration version of NutriBalance are provided in **Supplementary Material**. It should be noted that the demo version includes a list of fertilizers for demonstration purposes, which can be modified by the user.

### 2.4 Evaluation of the system performance

#### 2.4.1 Technical assessment

The objective was to evaluate the system’s capacity to perform a specific and precise injection of fertilizers for different concentrations and water quality conditions. To that end, six trials were carried out in 2021 and 2022, and covered periods in which the crop had different nutritional requirements.

##### 2.4.1.1 Irrigation water

Two sources of water were available at the farm: (i) DSW from the coastal desalination plant of Escombreras (30 km from the farm); and (ii) fresh water provided by the Tagus-Segura Water canal (FW). For the trial, both sources were used alone and as a blend in equal proportions of both sources (MW = 50% DSW + 50% FW). **Table 1** shows the physical and chemical properties and the price of the water sources used for the trial.

**Table 1:** Average price and values of physical and chemical properties of the water sources used for the trial.

Source	Price (€/m^3)^	pH	EC (dS/m)	Cl^-^ (mg/L)	NO_3_^-^ (mg/L)	PO_4_^3-^(mg/L)	NH_4_^+^ (mg/L)	K^+^ (mg/L)	Ca^2+^ (mg/L)	Mg^2+^ (mg/L)	Na^+^ (mg/L)	B (mg/L)
DSW	0.60	8.3 ± 0.2	1.0 ± 0.1	315.9 ± 63.7	2.6 ± 1.0	2.9 ± 2	0.0 ± 0.0	8.2 ± 0.8	35.1 ± 12.4	12.5 ± 8	190.2 ± 38.5	0.9 ± 0.1
MW	0.48	8.0 ± 0.1	1.2 ± 0.2	261.5 14.9	4.1 ± 0.9	2.9 ± 2.0	0.0 ± 0.0	7.2 ± 0.8	49.4 ± 15.1	20.8 ± 8.9	154.5 ± 18.6	0.6 ± 0.1
FW	0.35	7.7 ± 0.2	1.4 ± 0.2	187.0 ± 38.5	5.4 ± 1.7	3.8 ± 1.2	0.0 ± 0.0	6.4 ± 2.4	63.5 ± 12.8	32.2 + 9.2	117.9 ± 27.9	0.4 ± 0.1

Averaged values from 24 samples taken in 2021 and 2022.

##### 2.4.1.2 Fertilizers

The macro- and micro-nutrients were supplied with fertilizers commonly used in the orchards of the study area and acquired from local suppliers. **Table 2** shows the fertilizers used for the trial, their prices, and their main physical-chemical characteristics.

**Table 2:** Average price and main properties of fertilizers used in the trial.

Commercial fertilizer	Chemical formula	N−P−K + Ca+ Mg richness	Molecular mass (g/mol)	Density (g/L or kg/m^3^*)	Price (EUR/L or EUR/kg*)
Phosphoric acid	H_3_PO_4_	0−52.5−0	98.00	1580	0.66
Potassium nitrate	KNO_3_	13.5−0−46.2	101.10	2110	0.88
Ammonium nitrate	NH_4_NO_3_	34.5−0−0	80.04	1720	0.36
Magnesium nitrate	Mg(NO_3_)_2_	7−0−0 + 9.5 MgO	256.41	2300	0.57
Calcium nitrate	Ca(NO_3_)_2_	15−0−0 + 26 CaO	164.09	2500	0.44
Nitric acid	HNO_3_	12−0−0	63.00	1325	0.41
Unicquel (Iron chelate)	–	Fe 6%	–	460*	6.90*
Copper shuttle	–	Cu 6.13%	–	1287	8.50
Vitasève (Biostimulant with micronutrients)	–	MgO 5% + Mo 0.1% + B 0.2% + Mn 0.5% + Zn 0.5%	–	1210	9.00

The symbol – is normally used to link the quantity of nitrogen (N), phosphorus (P) and potassium (K) per liters or kilograms provided by a complex fertilizer which supply N, P and K simultaneously. It is usually specified in the technical datasheet of the fertilizer.

##### 2.4.1.3 Crop nutritional requirements

In each of the trials, the values of nutritional requirements and the fertilizers used varied depending on the tree age and stage. **Table 3** shows the nutrient requirements per month during the experimental period. The fertilizers KNO_3_, NH_4_NO_3_ and H_3_PO_4_, were used for trials 1 - 3, whereas Ca(NO_3_)_2_ and Mg(NO_3_)_2_ were used for trials 4 - 6. For each trial, DSW and FW sources were used alone and as a blend in equal proportions (MW).

**Table 3:** Monthly nutrient requirements for *Citrus x paradisi* (var. Rio Red) recommended by the field technician.

Crop	Month	N (kg/ha)	P_2_O_5_ (kg/ha)	K_2_O (kg/ha)	CaO (kg/ha)	MgO (kg/ha)	Cu (kg/ha)	Fe (kg/ha)
1−2 years of age (2021)	January		0.03	0.43				
	February	1.67	0.07	0.98				0.37
	March	2.62	0.10	1.50				0.55
	April	3.89	0.07	1.28	1.98	0.88		
	May	4.45	0.07	1.57	2.22	0.99		
	June	7.70	0.13	2.54	3.92	1.75	0.39	
	July	12.93	0.22	4.27	6.59	2.94		
	August	5.48	0.82	3.39				
	September	4.98	0.13	1.84				0.29
	October	2.74	0.13	1.69				0.30
	November		0.06	0.84				
	December		0.05	0.59				
	TOTAL	46.46	1.87	20.91	14.71	6.57	0.39	1.51
3−4 years of age (2022)	January		0.30	1.64				
	February	5.22	0.59	3.71				0.49
	March	8.16	0.87	5.72				0.74
	April	12.13	0.59	4.86	5.61	1.57		
	May	13.89	0.67	5.97	6.29	1.76		
	June	24.03	1.18	9.66	11.11	3.11	0.64	
	July	40.35	1.97	16.23	18.65	5.23		
	August	17.09	7.46	12.89				
	September	15.52	1.18	6.98				0.39
	October	8.54	1.17	6.44				0.41
	November		0.58	3.21				
	December		0.48	2.23				
	TOTAL	144.92	17.04	79.55	41.66	11.68	0.64	2.04

It is important to highlight that the values in **Table 3** differ from the recommendations for Star Ruby and Rio Red grapefruit varieties found in the literature (Wiedenfeld et al., 2009; Legaz et al., 2010; Pérez-Pérez et al., 2015). These differences are mainly due to the fact that the farm is located in a Nitrates Vulnerable Zone, where farmers must comply with fertilizer control laws (Royal Decree 261/1996, Law 3/2020 of recovery and protection of Mar Menor, and Order 12/2019 of Nitrates Vulnerable Zones).

##### 2.4.1.4 Sampling method and lab analyses

The sampling tank collected a total irrigation volume (water + fertilizer) of 1 m^3^ for each test. Samples of 0.5 L per test (1 type of water + 1 fertilization prescription) were collected in glass bottles and transported in an icebox to the laboratory. They were stored at 5 °C before being processed for physical and chemical analyses.

The EC of the water was measured with a conductivity instrument GLP-31 (Crison Instruments S.A., Barcelona, Spain), and the pH was measured with a pH-meter GLP-21 (Crison Instruments S.A., Barcelona, Spain). An inductively coupled plasma (ICP-MS Agilent Technologies, Model 7900, Santa Clara, CA, USA) was used to determine the concentrations of Na^+^, K^+^, NH_4_^+^, Ca^2+^ and Mg^2+^. Anions (Cl^-^, NO_3_^-^, PO_4_^3-^ and SO_4_^2-^) were quantified by ion chromatography with liquid chromatograph (Thermo Scientific Dionex, Model ICS-2100, Thermo Scientific, Basel, Switzerland).

The concentrations measured in the samples were compared to the concentrations obtained theoretically with the NutriBalance program in order to assess the system’s ability to accurately provide different concentrations of fertilizers with varying feed waters.

##### 2.4.1.5 Statistical analysis

The statistical analysis performed was a weighted analysis of variance (ANOVA) using the statistical software IBM SPSS Statistics v. 21. The significance level used was *p* ≤ 0.05.

#### 2.4.2 Economic assessment

The following costs were considered for the economic evaluation: (i) the depreciation cost of the equipment (fixed cost) and (ii) the fertilizer and water consumption cost (variable cost). The cost of the energy used and the maintenance costs of the equipment were not included. The average flow of the experimental system was 1.2 m^3^/h (D1). Since that flow is substantially lower than those of commercial systems, the calculations were also made for analogous irrigation heads with flows of 12 m^3^/h (D2) and 28 m^3^/h (D3). These flows offer more standard and realistic scale results. The systems D1−D3 could irrigate 0.28, 1.00, and 3.18 ha of citrus crop, respectively (*i.e.*, approximately 144, 540, and 1720 citrus trees).

##### 2.4.2.1 Depreciation costs

The amortization cost was calculated as the corresponding annual payment, as follows:


(7) $$C=\;V_{ad}\cdot \frac{i\cdot {\left(1+i\right)}^{a}}{{\left(1+i\right)}^{a}-1}$$

where *C* (EUR/year) was the annual payment fee (EUR), V*_ad_* the equipment acquisition value (EUR), *i* the interest (%), and *a* the equipment lifespan (years).

The lifespan of the system was estimated at 12 years, considering 1224 h of annual operation, and the interest rate used was 5%. The acquisition value of the D1 experimental system provided by the manufacturer was 18,720 EUR. For commercial size systems, the values provided were 25,230 EUR and 34,550 EUR, for D2 (12 m^3^/h) and D3 (28 m^3^/h), respectively.

##### 2.4.2.2 Fertilizer and water cost

The prices of water and fertilizers during the experimental work (2021−2022) are given in **Tables 1**, **2**, respectively. The estimated yearly prices for the period 2018−2030 are also provided in **Supplementary Material** (see **Supplementary Table S1**) to assess the potential savings over the lifespan of the equipment, in accordance with fertilizer price trends.

##### 2.4.2.3 Estimation of potential savings

In order to consider the potential savings from the use of NutriBalance, the consumption of fertilizers per hectare was calculated for two scenarios:

(i) Without NutriBalance: Based on the fertilizer supply calculated by the field technician, which was commonly used in the commercial farm where the trial was conducted.
(ii) With NutriBalance: Based on the fertilizer supply calculated by NutriBalance to fulfill the nutritional requirements of the crop at the most profitable cost.

In order to estimate the potential savings for the lifespan of the equipment (12 years), two cases were considered:

(i) Case 1, assuming that the fertilizer price remained steady (**Table 2**).
(ii) Case 2, in which the fertilizer price continued to increase following current trends (**Table S1**).

In addition, since the trees of the experimental site were three years old at the end of the trial, an increase in fertilizer consumption of 15% per year was considered until the beginning of adulthood of the trees (seven years of age), after which fertilizer consumption is assumed to remain almost steady (Maestre-Valero et al., 2016).

## 3 Results

### 3.1 Technical assessment

The fertigation system was observed to provide the calculated fertigation solution accurately. Overall, all the trials showed the great ability (error below 7% for most ions) of the system to provide the prescribed fertigation dose with different combinations of irrigation water. These results evidenced the good interoperability between NutriBalance and the irrigation head. In addition, it should be noted that the system provides a user-friendly interface and that the settings can be adapted to the user’s particular case (demo version link in the **Supplementary Material**).

**Figure 3:**
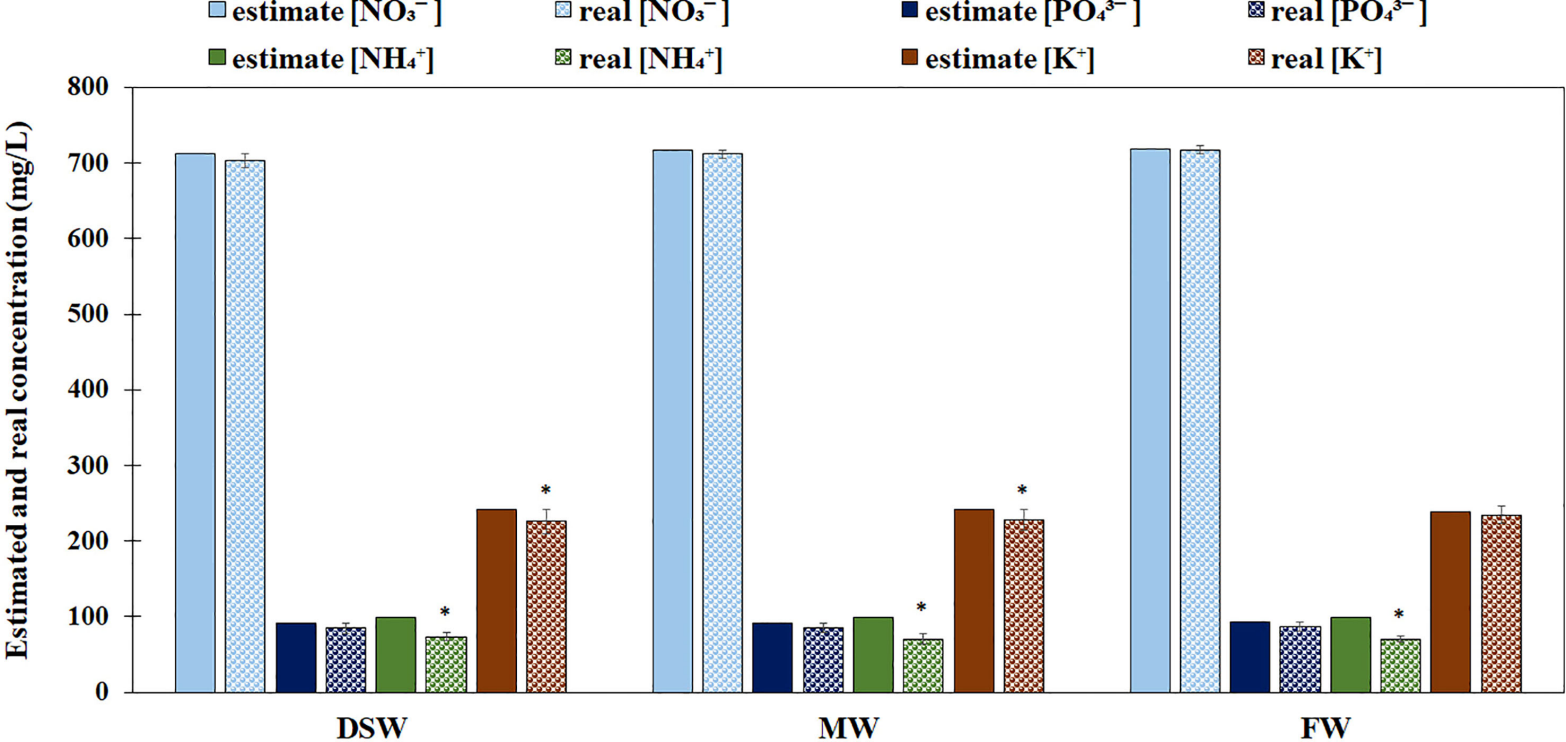
Estimated and measured concentrations in trial 3. Asterisks indicate significant differences (*p* ≤ 0.05). The fertilizers KNO3, NH4NO3, and H3PO4 were used for the trial.

**Figure 4:**
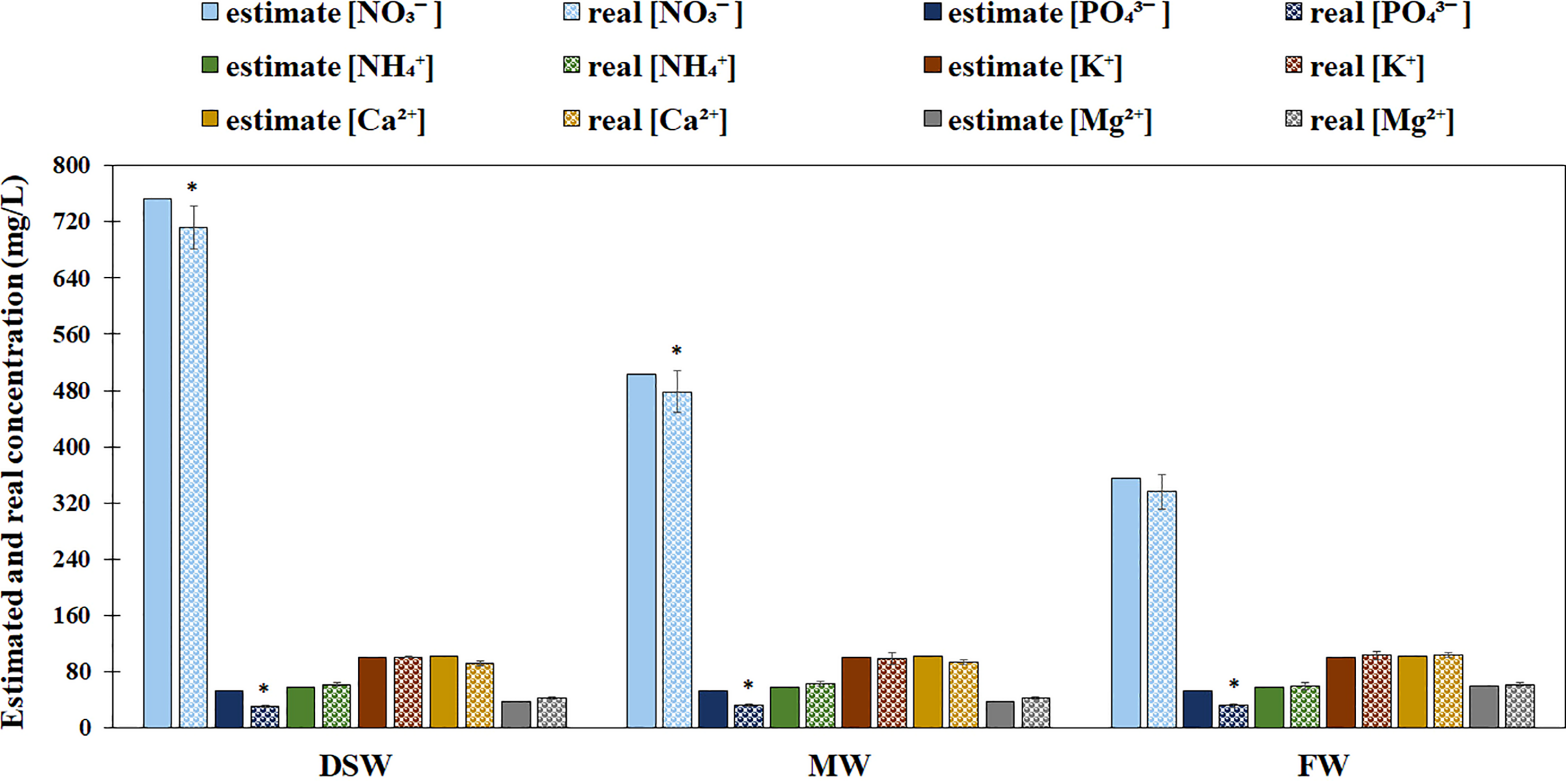
Estimated and measured concentrations in trial 6. Asterisks indicate significant differences (*p* ≤ 0.05). The fertilizers KNO3, NH4NO3, H3PO4, Ca(NO_3_)_2_, and Mg(NO_3_)_2_ were used for the trial.

**Figures 3**, **4** show the comparison between the ion concentrations computed by NutriBalance and the real concentrations supplied (measured in the samples), in trials 3 and 6, respectively. These trials were selected for the demonstration, since trial 3 corresponded to the appearance of sprouting buds and required lower amounts of fertilizer, and trial 6 corresponded to fruit development and required a relatively high dose of fertilizer. The rest of the trial results are presented in **Supplementary Material**.

The results from both trials (3 and 6) showed very good agreement between the estimated and measured concentrations. Errors in trial 3 ranged from 0.1 to 6.7%, with the exception of NH_4_^+^, for which the error ranged from 26.0 to 29.5% (**Figure 3**). In trial 6, in which five different types of fertilizers were used, no significant differences were observed for most ions (**Figure 4**). The exception in this case were NO_3_^-^ and PO_4_^3-^, with errors of up to 25.0%. The rest of the trials (except trial 1) presented very similar results (**Figures S4**−**S7**). In trial 1, some undiluted fertilizers were used, causing some significant nutritional variations in the measurements compared to the calculated values. In the rest of the trials, the fertilizers were diluted in the tanks and more precise injection was achieved. This highlights the importance of the dilution of the fertilizers and the mechanical adjustments of the rotameters to achieve good results. Moreover, unexpected concentrations of Ca^2+^ and Mg^2+^ ions were found in the samples of trials 1 - 3 (data not shown), despite the fact that only the fertilizers KNO_3_, NH_4_NO_3_ and H_3_PO_4_ were used. The fertilizers may have contained amounts of unreported ions, which may, to a certain extent, have led to a slight variation in the rest of the ions.

### 3.2 Economic assessment

**Table 4** shows the total costs and potential savings with vs. without NutriBalance, for the three designs (D1−D3), and different feed waters (DSW, FW, MW). The most relevant finding was that fertilizer consumption was significantly reduced when using NutriBalance. In particular, the fertilizer cost without NutriBalance for D2 was 342.01 EUR/ha/year for 2021 and 687.93 EUR/ha/year for 2022, while it decreased by 41.7% (142.69 EUR/ha/year), and 40.9% (281.39 EUR/ha/year), for 2021 and 2022, respectively, when it was used.

**Table 4:** Depreciation, water and fertilizer costs for D1, D2, and D3 irrigation systems.

	Without NutriBalance				With NutriBalance					
Year	Fertilization	Irrigation water*	Depreciation**	Total cost	Fertilization	Irrigation water	Depreciation	Total cost	Cost saving per year	Water source
EUR/year										
D1 (0.28 ha)										
2021	94.81	343.74	2112.09	2550.64	56.06	432.36	2112.09	2600.51	-49.87	DSW
					55.31	343.74	2112.09	2511.15	39.49	MW
					54.39	255.12	2112.09	2421.60	129.04	FW
2022	190.69	415.95	2112.09	2718.74	114.82	523.19	2112.09	2750.10	-31.36	DSW
					113.30	415.95	2112.09	2641.34	77.39	MW
					109.96	308.72	2112.09	2530.76	187.97	FW
D2 (1.0 ha)										
2021	342.01	1240.05	2846.65	4428.72	202.23	1559.75	2846.65	4608.64	-179.92	DSW
					199.55	1240.05	2846.65	4286.25	142.47	MW
					196.20	920.36	2846.65	3963.21	465.51	FW
2022	687.93	1500.55	2846.65	5035.13	414.21	1887.40	2846.65	5148.26	-113.13	DSW
					408.73	1500.55	2846.65	4755.93	279.20	MW
					396.67	1113.69	2846.65	4357.02	678.11	FW
D3 (3.18 ha)										
2021	1087.60	3943.37	3898.02	8928.99	643.10	4960.01	3898.02	9501.13	-572.14	DSW
					634.56	3943.37	3898.02	8475.95	453.04	MW
					623.92	2926.73	3898.02	7448.68	1480.31	FW
2022	2187.62	4771.74	3898.02	10857.37	1317.18	6001.93	3898.02	11217.13	-359.76	DSW
					1299.77	4771.74	3898.02	9969.53	887.85	MW
					1261.42	3541.54	3898.02	8700.98	2156.39	FW

(^*^) Irrigation water cost was calculated considering MW source for irrigation water. (^**^) Note that the depreciation cost was calculated as an annual payment fee for 12 years of operation.

The cost of the water was calculated using the water prices paid during the experimental work, which were 0.35 EUR/m^3^ for FW and 0.60 EUR/m^3^ for DSW. The irrigation water cost in 2021 for the D2 design ranged from 1559.75 EUR/ha/year for DSW irrigation to 920.36 EUR/ha/year for FW irrigation. Such values increased by 20.7% in 2022; *i.e*., 1887.4 and 1113.67 EUR/ha/year (**Table 4**), due to crop growth.

The fixed cost of the system (i.e., depreciation cost) depended mainly on the size of the system, particularly the pumping group, the electrical devices and the water conduction elements. D1 had a depreciation cost of 1.92 EUR/m^3^ (2112.09 EUR/year), whereas D2 and D3 had a much lower depreciation cost of 0.19 EUR/m^3^ (2846.65 EUR/year) and 0.11 EUR/m^3^ (3898.02 EUR/year), respectively, highlighting the importance of the scale factor.

**Figure 5:**
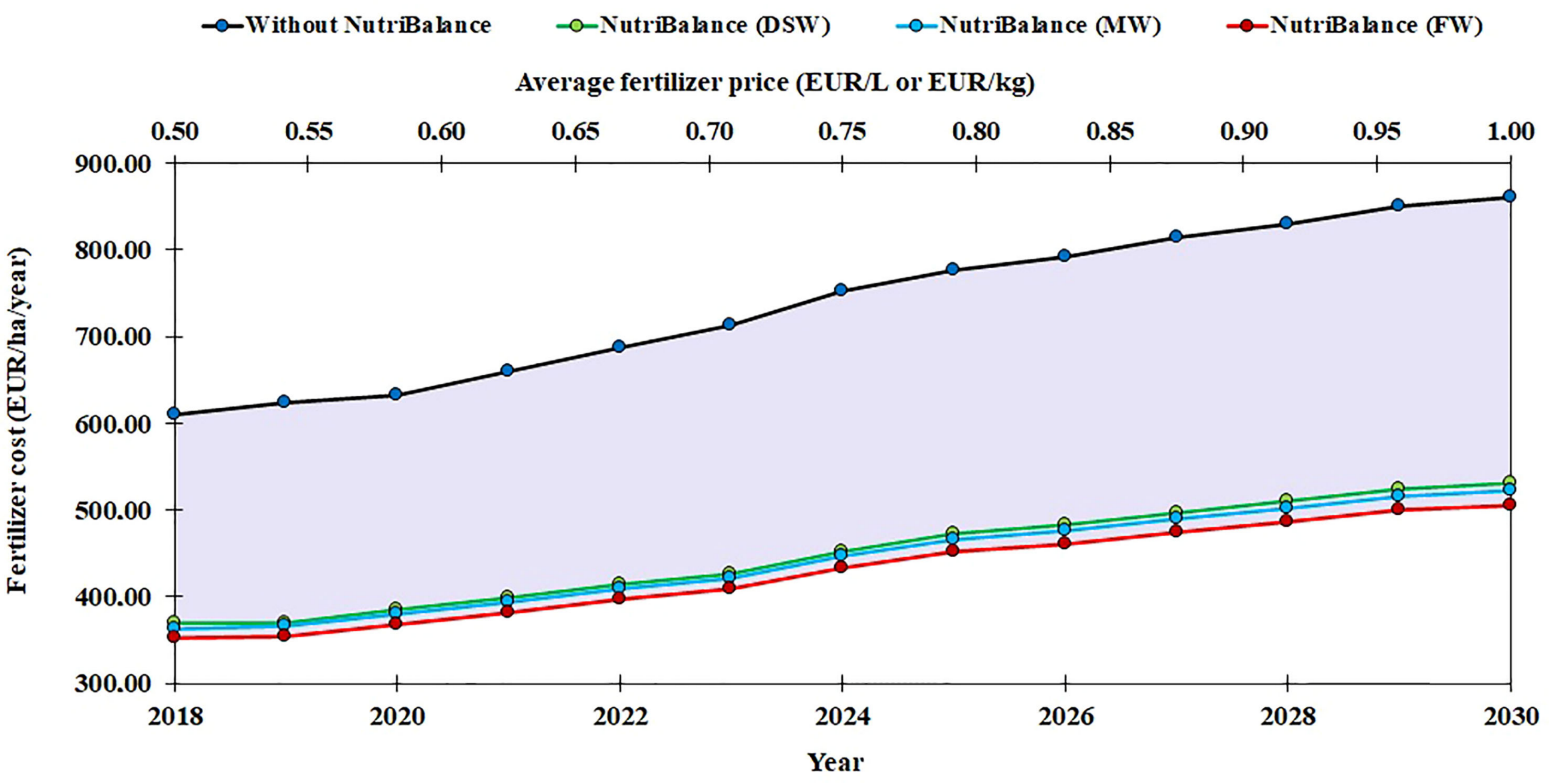
Projected increase in fertilizer cost savings considering an average increase in fertilizer prices. The shaded area represents the costs savings when using NutriBalance with each type of irrigation water.

The total cost for the base scenario with D2 was 4428.72 EUR/ha/year for 2021 and 5035.13 EUR/ha/year for 2022, while it was 4286.03 EUR/ha/year and 4753.74 EUR/ha/year for 2021 and 2022, respectively, when NutriBalance was used. For the year with the highest estimated fertilizer cost (3-year-old grapefruit orchard, 2022), **Figure 5** shows how the implementation of NutriBalance can potentially save up to 340.60 EUR/ha/year. It is to be expected that the fertilizer savings will increase as the crop reaches an adult stage and requires more fertilizer. The water cost will also continue to increase moderately as the trees approach adulthood and also due to water price rises driven by the pressure on water resources.

**Figure 6:**
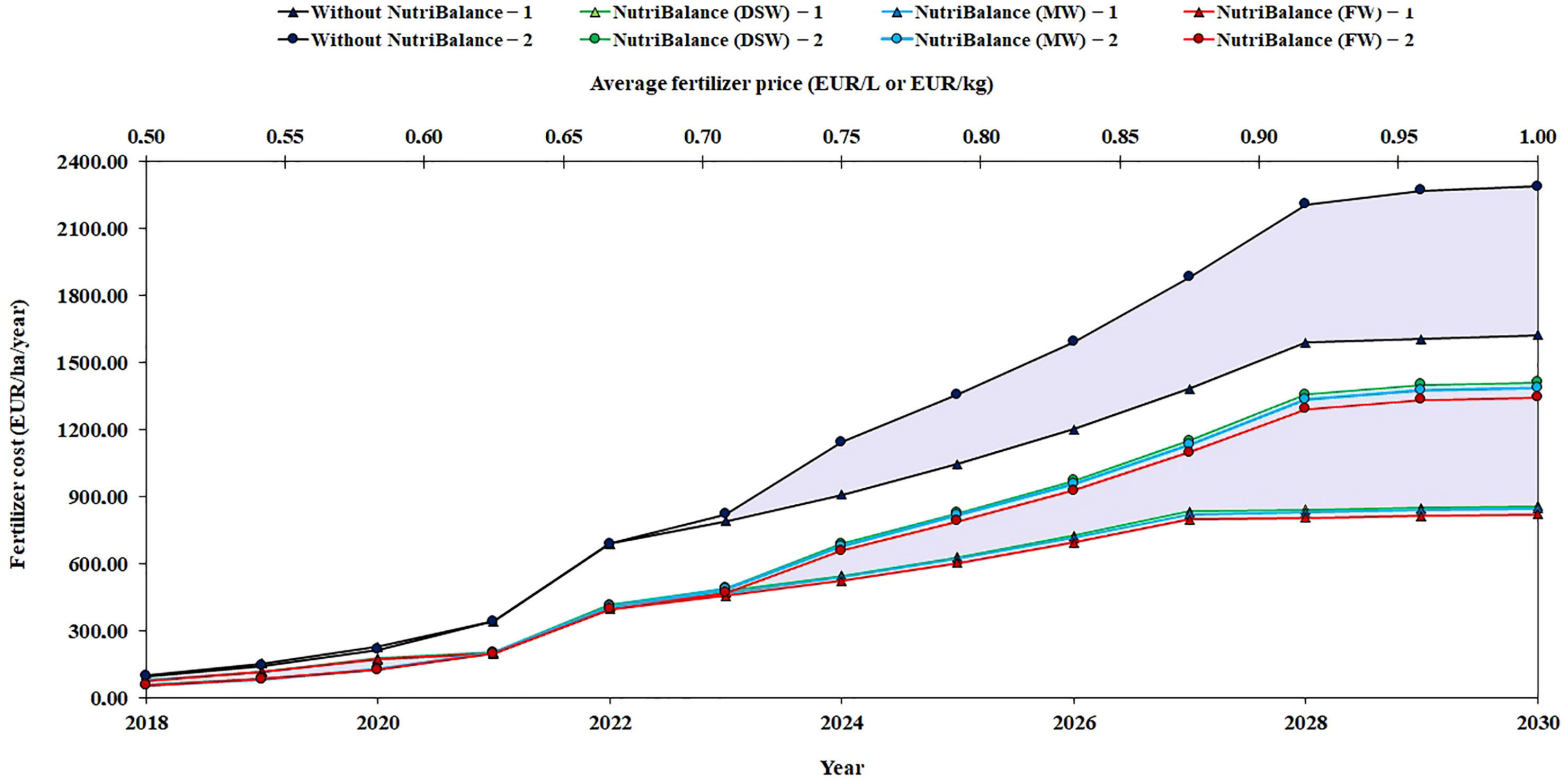
Evolution of orchard fertilizer cost with constant (1) and increasing (2) fertilizer price. The shaded area represents the increase in fertilizer cost due to the rising price trend.

The potential savings during the lifespan of the equipment (12 years) were calculated with constant and increasing fertilizer prices (**Figure 6**). Assuming a quasi-linear behavior of the cost savings and the increase in fertilizer prices during the lifespan of the equipment, the savings for the orchard irrigated with MW would amount to 6008.61 EUR (500.72 EUR/ha/year). Considering that the implementation of NutriBalance in a high-tech irrigation head is estimated to cost around 150 EUR/ha/year (supplier’s estimate), it would appear to be an interesting investment for the studied scenarios.

## 4 Discussion

### 4.1 Technical functionality

The trials of this study showed the high accuracy of a readily available high-tech head to provide the crop with the exact and most profitable fertilizer dosing calculated by the NutriBalance program. The main advance of the system is the ability to adapt the fertilizer dosing to different feed waters, rather than limiting the calculation to preset values of water quality (Pagán et al., 2015; Bueno-Delgado et al., 2016; Pérez-Castro et al., 2017).

Notwithstanding the good performance of the studied system, it is important to pay attention to the relationship between different fertilizers in order to achieve the best result. The highest divergence between measurements and calculations was found when DSW was used as the feed water. It has a low mineral content and requires more fertilizer inputs, and hence, presumably had higher interactions between fertilizers. In fact, the interactions of these elements in the formulation or mixing have been reported to interfere with the dynamics of these nutrients during fertigation (Li et al., 2019), although all the mechanisms involved in the process have yet to be investigated (Grohskopf et al., 2019). In crop fertilization, there is frequently a concomitant supply of Ca, P, and N, from different fertilizers or even in the same complex fertilizer. The effect of the interaction between calcium (Ca) and nitrate or phosphorus (P) and nitrogen (N) in fertilizers can have a notable significance (Grohskopf et al., 2019; Li et al., 2019; Luo et al., 2019).

### 4.2 Reduction of fertilizer consumption and system profitability

The results of this trial show that the studied system can reduce the fertilization cost by between 41.7 and 40.9% for two- and three-year-old grapefruit trees, respectively. The implementation of NutriBalance has been shown to potentially offer savings of up to 340.60 EUR/ha/year for a three-year-old orchard. Savings are expected to increase as the crop reaches an adult stage and demands more fertilizer. Moreover, our mid-term estimations show savings that exceed 500 EUR/ha/year, if the fertilizer price continues its current upward trend (**Figure 5**).

Our findings are in agreement with the potential savings expected from the use of other fertilizer cost optimization tools such as Optifer (Pagán et al., 2015) and Ecofert (Bueno-Delgado et al., 2016), which were tested for horticultural crops. It should be noted that those tools considered the amount of nutrients supplied by the irrigation water, like NutriBalance. This is particularly relevant when using non-conventional waters with varying nutrient content (Maestre-Valero et al., 2019).

Concerning the influence of the type of irrigation water, the present work shows how the fertilization cost increases when integrating DSW for irrigation, due to its lower nutrient content. Martínez-Alvarez et al. (2020) calculated the increases in water and fertilizer costs of totally (100%) and partially (50%) replacing conventional irrigation water with DSW for the most representative crops of south-eastern Spain. In the case with 100% DSW, the fertilization cost was estimated to increase by over 25% for a lemon citrus crop in the adult stage. In our case, for two- and three-year-old grapefruit trees it increased the total cost by 14.00 and 15.40%, respectively (**Table 4**). Therefore, the increase for adult grapefruit trees could be in excess of 20%. Considering this, and in the current context of freshwater scarcity, which is expected to boost DSW consumption (Martínez-Alvarez et al., 2020), then fertilizer optimization consumption systems become even more useful to preserve profitability (Gallardo et al., 2020; Seethalakshmi et al., 2021).

## 5 Conclusions

This work has experimentally assessed the technical and economic viability of a smart fertigation system. It consisted of a readily available high-tech irrigation head in which the novel fertigation optimization program named NutriBalance was implemented. The trials performed have shown (i) the good interoperability between NutriBalance and the irrigation head, (ii) the nearly flawless ability of the system to provide the computed fertigation prescription with different combinations of DSW and FW feed water, and (iii) the great potential of NutriBalance to curb fertilizer costs.

The overall performance of the system was highly satisfactory, with errors between the prescribed and applied nutrients of less than 7% for most ions. However, errors of up to 30% were observed in some of the tests for NH_4_^+^, NO_3_^-^ and PO_4_^3-^, with the highest errors observed when only DSW was used. This highlights the importance of accounting for possible interactions between fertilizers, particularly when the feed water has a lower mineral content and more fertilizer is required.

The implementation of NutriBalance can potentially reduce the fertilizer cost for a three-year-old grapefruit orchard by over 40% (340 EUR/ha/year). Considering the upward trend of fertilizer prices, savings during the lifespan of the equipment (12 years) have been estimated to reach around 500 EUR/ha/year. The latter figure is substantially lower than the estimated cost of implementing the smart tool in the irrigation head (around 150 EUR/ha/year), evidencing the economic feasibility of the system.

The study details the good interoperability between smart programs and available (and affordable) high-tech irrigation heads. The positive results of this experimental study are expected to encourage the adoption of smart systems into the conventional agricultural model. It is recommended that users account for potential interactions between fertilizers for a given feed water for optimal performance of the system. This is particularly relevant when using new complex fertilizers and biostimulant formulations. Future improved versions of NutriBalance will be able to warn the user about possible interactions and allow updates with the most recent findings on this aspect.

## Data availability statement

The original contributions presented in the study are included in the article/**Supplementary Material**. Further inquiries can be directed to the corresponding author.

## Author contributions

Conceptualization: JM. Data curation: AIM and JFMV. Formal analysis: AIM. Funding acquisition: JFMV and BGE. Investigation: All authors. Methodology: AIM, JFMV, VMA, FJGG and BGE. Project administration: JFMV. Writing−original draft: AIM. Writing−review and editing, AIM, JFMV, VMA, FJJC and BGE. All authors contributed to the article and approved the submitted version.

## Supplementary material

Trial version link of NutriBalance
https://nutrimac.nutricontrol.com/nutribalance/#/.

Supplementary Figure 1 Specific information provided by the user.

Supplementary Figure 2 Registered and available fertilizers.

Supplementary Figure 3 Crop nutritional requirements.

Supplementary Figure 4 Estimated and measured concentration in trial 1. Asterisks indicate significant differences (*p* ≤ 0.05). The fertilizers KNO_3_, NH_4_NO_3_ and H_3_PO_4_ were used for the trial.

Supplementary Figure 5 Estimated and measured concentration in trial 2. Asterisks indicate significant differences (*p* ≤ 0.05). The fertilizers KNO_3_, NH_4_NO_3_ and H_3_PO_4_ were used for the trial.

Supplementary Figure 6 Estimated and measured concentration in trial 4. Asterisks indicate significant differences (*p* ≤ 0.05). The fertilizers KNO_3_, NH_4_NO_3_, H_3_PO_4_, Ca(NO_3_)_2_ and Mg(NO_3_)_2_ were used for the trial.

Supplementary Figure 7 Estimated and measured concentration in trial 5. Asterisks indicate significant differences (*p* ≤ 0.05). The fertilizers KNO_3_, NH_4_NO_3_, H_3_PO_4_, Ca(NO_3_)_2_ and Mg(NO_3_)_2_ were used for the trial.

Supplementary Table 1 Evolution of yearly prices of inorganic fertilizers (EUR/L or EUR/kg).
