# Supplementary material for: Evaluation of an autonomous smart system for optimal management of fertigation with variable sources of irrigation water
Source: Front Plant Sci. 2023 Apr 12;14:1149956. doi: 10.3389/fpls.2023.1149956 (PMC10130640; doi:10.3389/fpls.2023.1149956)
Supplement: Trial version link of NutriBalance [file DataSheet_1.zip › Data Sheet 2/Data sheet 2.docx]

**Evaluation of an autonomous smart system for optimal management of fertigation with variable sources of irrigation water**

Alberto Imbernón-Mulero^1^*, José F. Maestre-Valero^1^, Victoriano Martínez-Alvarez^1^, Francisco J. García-García^2^, Francisco J. Jódar-Conesa^3^, Belén Gallego-Elvira^1^

^1^Department of Agricultural Engineering, Technical University of Cartagena, Paseo Alfonso XIII 48, 30203 Cartagena, Spain.

^2^Technical Direction, Nutricontrol S.L., Calle Bucarest, 26, 30353 Cartagena, Spain.

^3^Agrícola Conesa Martín S.L., Paraje Los Bastidas, 42, 30700 Torre Pacheco, Spain.

**^*^Correspondence:** Alberto Imbernón-Mulero **(**[alberto.imbernon@edu.upct.es](mailto:alberto.imbernon@edu.upct.es))

**The supplementary material includes Figures S1─S7 and Table S1**

Trial version link of NutriBalance: <https://nutrimac.nutricontrol.com/nutribalance/#/>

**
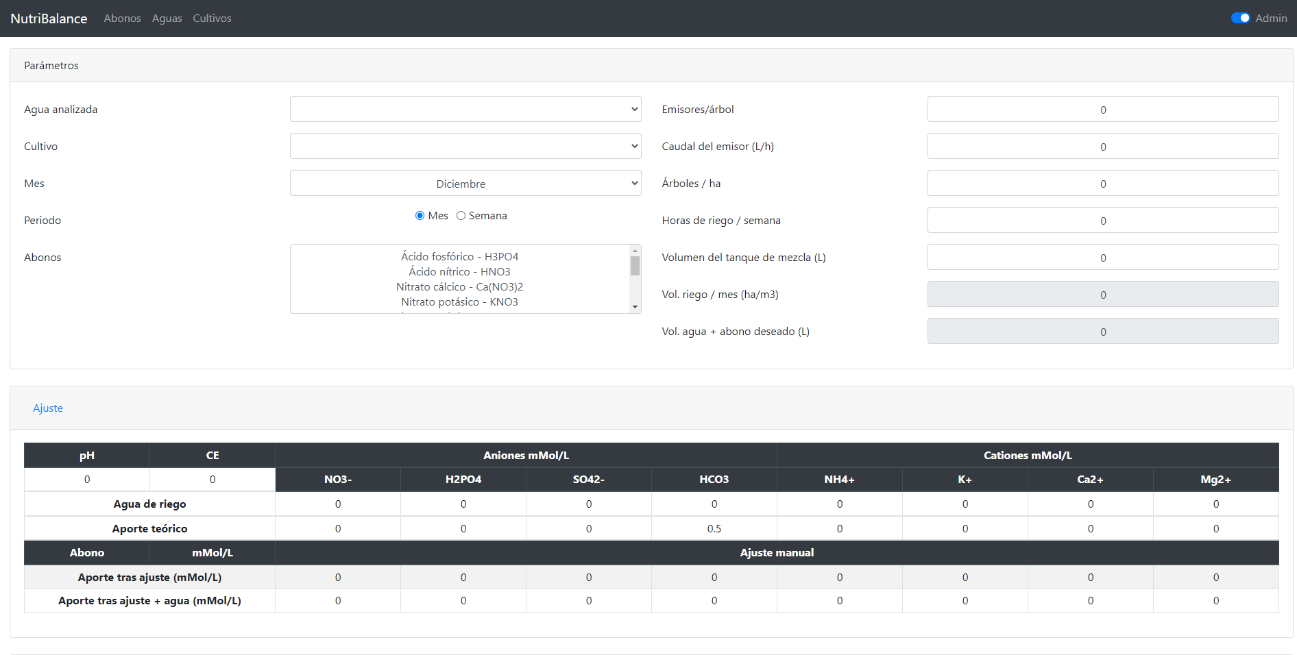
**

**Supplementary Figure S1.** Specific information provided by the user.

**
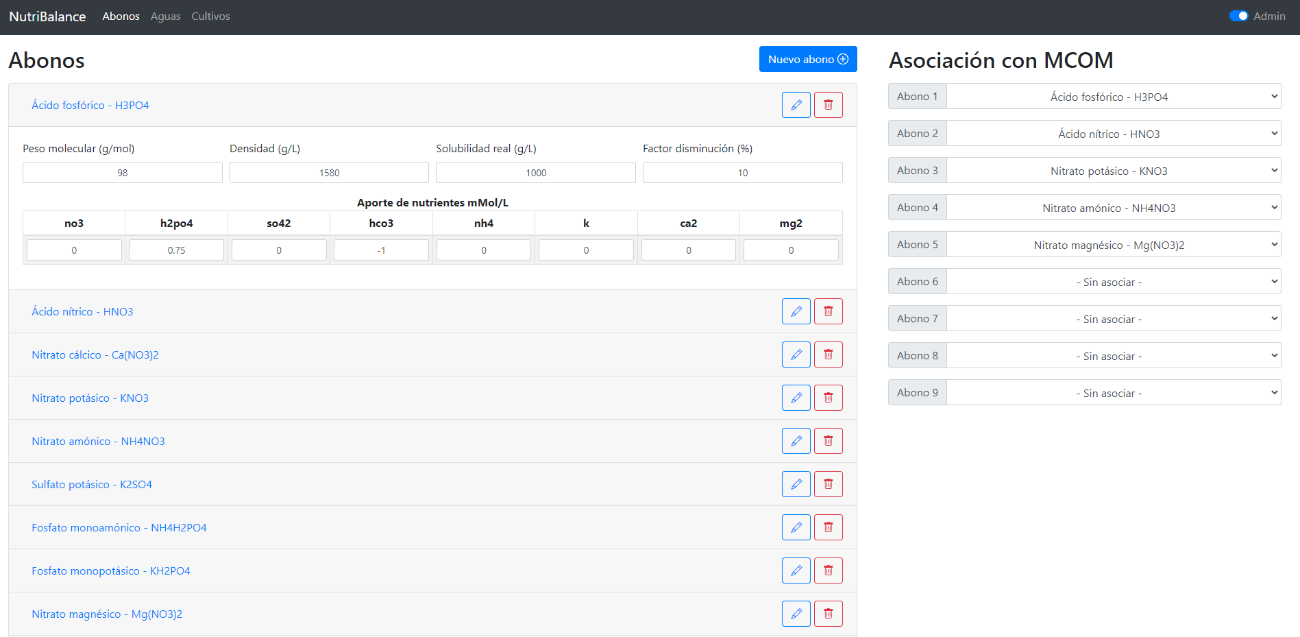
**

**Supplementary Figure S2.** Registered and available fertilizers.

**
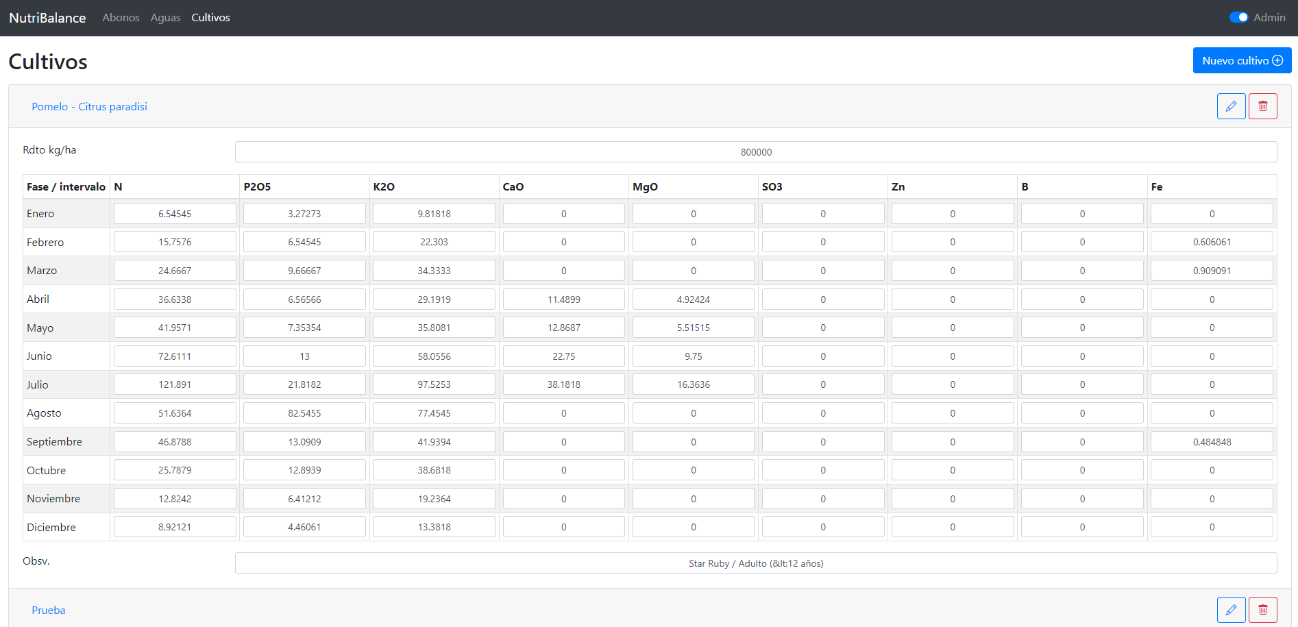
**

**Supplementary Figure S3.** Crop nutritional requirements.

**Supplementary Table S1.** Evolution of yearly prices of inorganic fertilizers (EUR/L or EUR/kg).

| **Year** | **H_3_PO_3_** | **K_2_SO_4_** | **KNO_3_** | **NH_4_NO_3_** | **HNO_3_** | **Mg(NO_3_)_2_** | **Ca(NO_3_)_2_** | **Copper shuttle** | **Vitasève** | **Unicquel (Iron chelate)** |
| --- | --- | --- | --- | --- | --- | --- | --- | --- | --- | --- |
| *2018* | 0.58 | 0.65 | 0.85 | 0.38 | 0.37 | 0.55 | 0.41 | 7.30 | 8.10 | 6.10 |
| *2019* | 0.62 | 0.67 | 1.03 | 0.35 | 0.38 | 0.55 | 0.42 | 7.40 | 8.20 | 6.00 |
| *2020* | 0.64 | 0.68 | 0.93 | 0.34 | 0.40 | 0.56 | 0.42 | 7.80 | 8.40 | 6.10 |
| ***2021*** | **0.66** | **0.68** | **0.88** | **0.36** | **0.41** | **0.57** | **0.44** | **8.50** | **9.00** | **6.50** |
| ***2022*** | **0.68** | **0.70** | **0.91** | **0.38** | **0.43** | **0.59** | **0.45** | **8.63** | **9.00** | **6.90** |
| *2023* | 0.71 | 0.73 | 0.95 | 0.39 | 0.44 | 0.62 | 0.47 | 8.84 | 9.27 | 7.17 |
| *2024* | 0.77 | 0.79 | 1.02 | 0.42 | 0.48 | 0.64 | 0.51 | 9.19 | 9.55 | 7.46 |
| *2025* | 0.79 | 0.81 | 1.04 | 0.45 | 0.52 | 0.66 | 0.55 | 9.23 | 9.84 | 7.53 |
| *2026* | 0.81 | 0.82 | 1.06 | 0.47 | 0.53 | 0.68 | 0.57 | 9.36 | 10.03 | 7.64 |
| *2027* | 0.84 | 0.85 | 1.10 | 0.49 | 0.54 | 0.69 | 0.59 | 9.52 | 10.23 | 7.82 |
| *2028* | 0.86 | 0.86 | 1.14 | 0.50 | 0.56 | 0.71 | 0.60 | 9.74 | 10.44 | 7.89 |
| *2029* | 0.89 | 0.88 | 1.20 | 0.51 | 0.59 | 0.73 | 0.62 | 9.89 | 10.54 | 7.96 |
| *2030* | 0.91 | 0.89 | 1.22 | 0.52 | 0.60 | 0.75 | 0.63 | 9.90 | 10.65 | 8.00 |

**
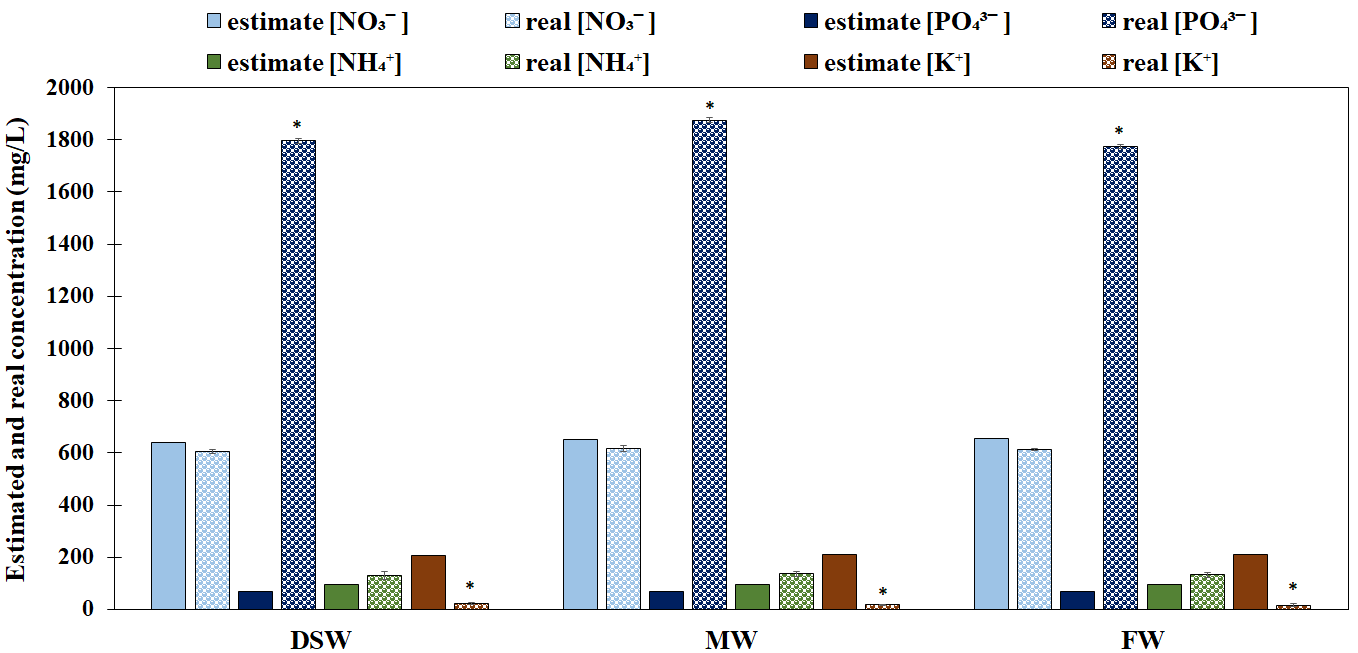
**

**Supplementary Figure S4**. Estimated and measured concentration in trial 1. Asterisks indicate significant differences (*p* ≤ 0.05). The fertilizers KNO_3_, NH_4_NO_3_ and H_3_PO_4_ were used for the trial.


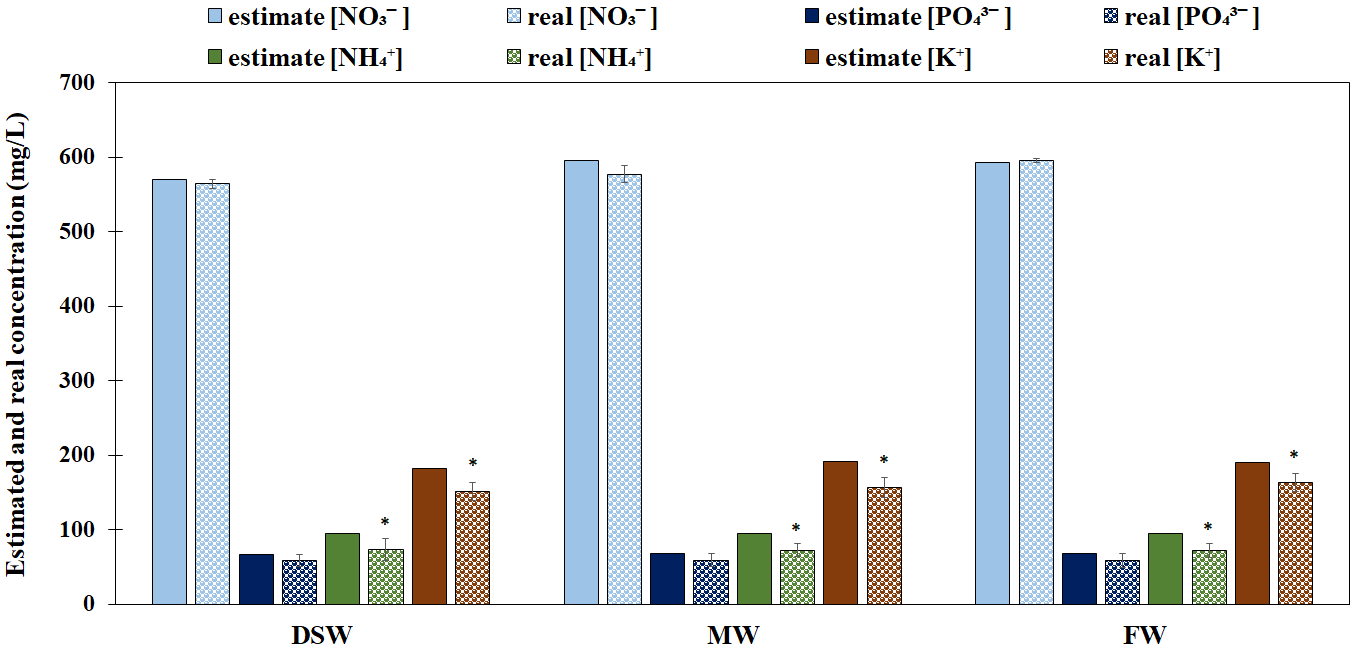


**Supplementary Figure S5**. Estimated and measured concentration in trial 2. Asterisks indicate significant differences (*p* ≤ 0.05). The fertilizers KNO_3_, NH_4_NO_3_ and H_3_PO_4_ were used for the trial.


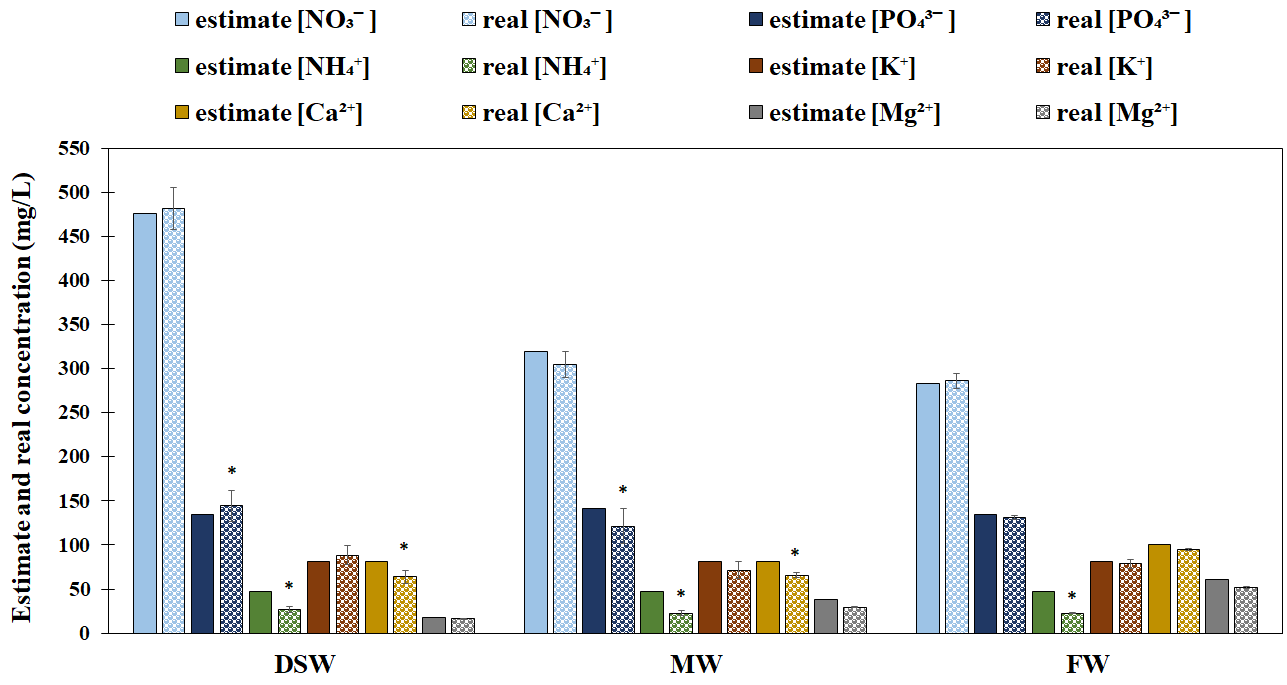


**Supplementary Figure S6**. Estimated and measured concentration in trial 4. Asterisks indicate significant differences (*p* ≤ 0.05). The fertilizers KNO_3_, NH_4_NO_3_, H_3_PO_4_, Ca(NO_3_)_2_ and Mg(NO_3_)_2_ were used for the trial.


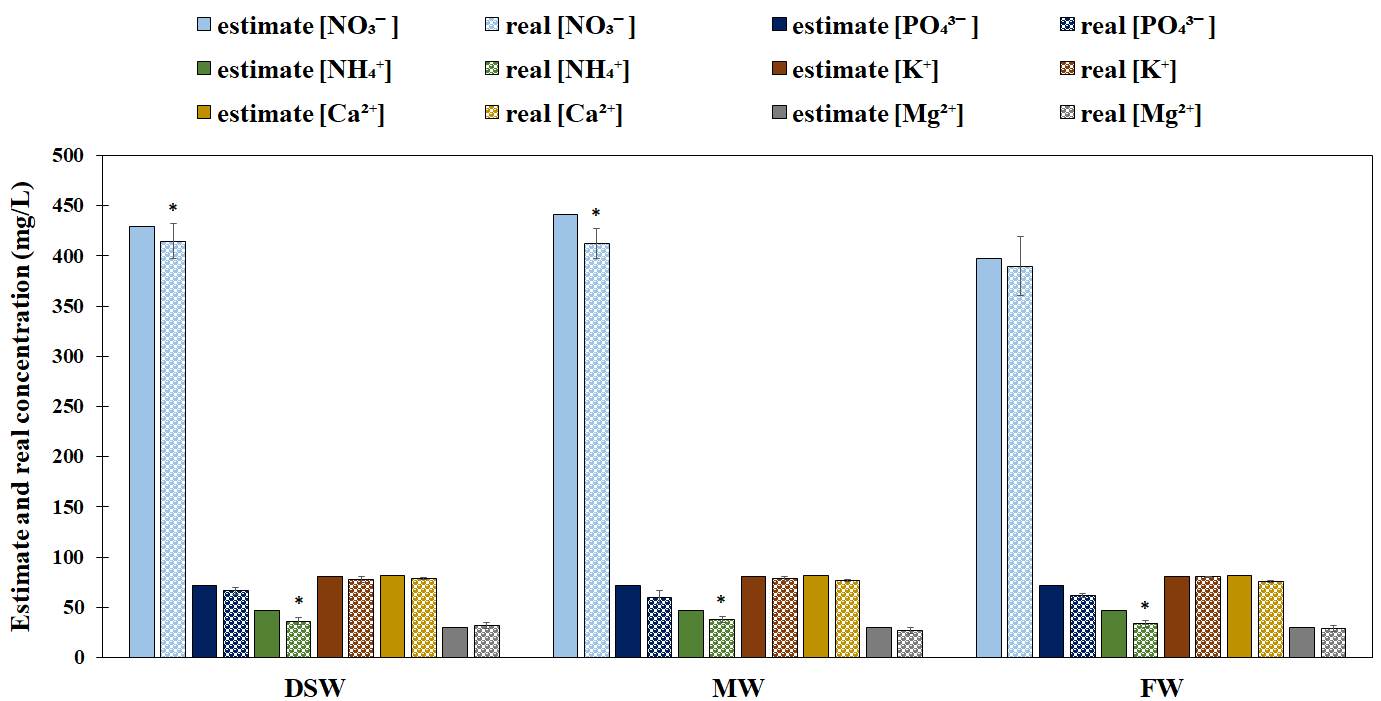


**Supplementary Figure 7**. Estimated and measured concentration in trial 5. Asterisks indicate significant differences (*p* ≤ 0.05). The fertilizers KNO_3_, NH_4_NO_3_, H_3_PO_4_, Ca(NO_3_)_2_ and Mg(NO_3_)_2_ were used for the trial.
