# Supplementary figures and images for: Evaluation of an autonomous smart system for optimal management of fertigation with variable sources of irrigation water
Source: Front Plant Sci. 2023 Apr 12;14:1149956. doi: 10.3389/fpls.2023.1149956 (PMC10130640; doi:10.3389/fpls.2023.1149956)

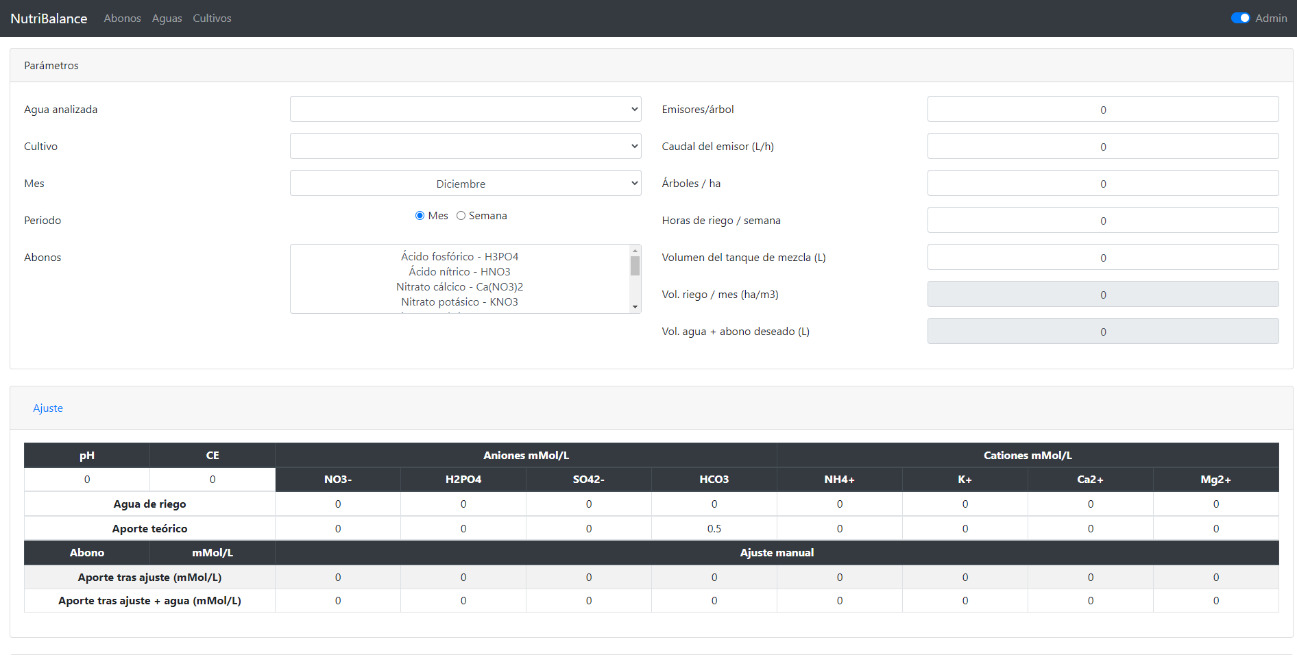

Supplement: Trial version link of NutriBalance [file DataSheet_1.zip › Data Sheet 2/Supplementary Figure S1.jpg]

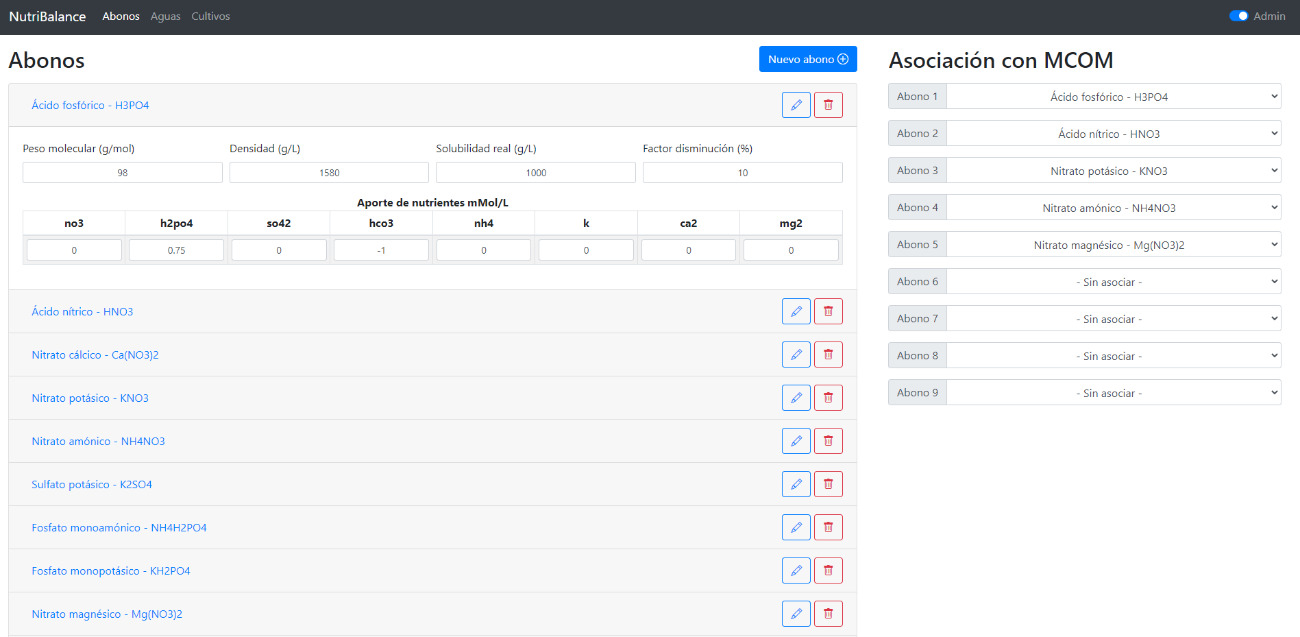

Supplement: Trial version link of NutriBalance [file DataSheet_1.zip › Data Sheet 2/Supplementary Figure S2.jpg]

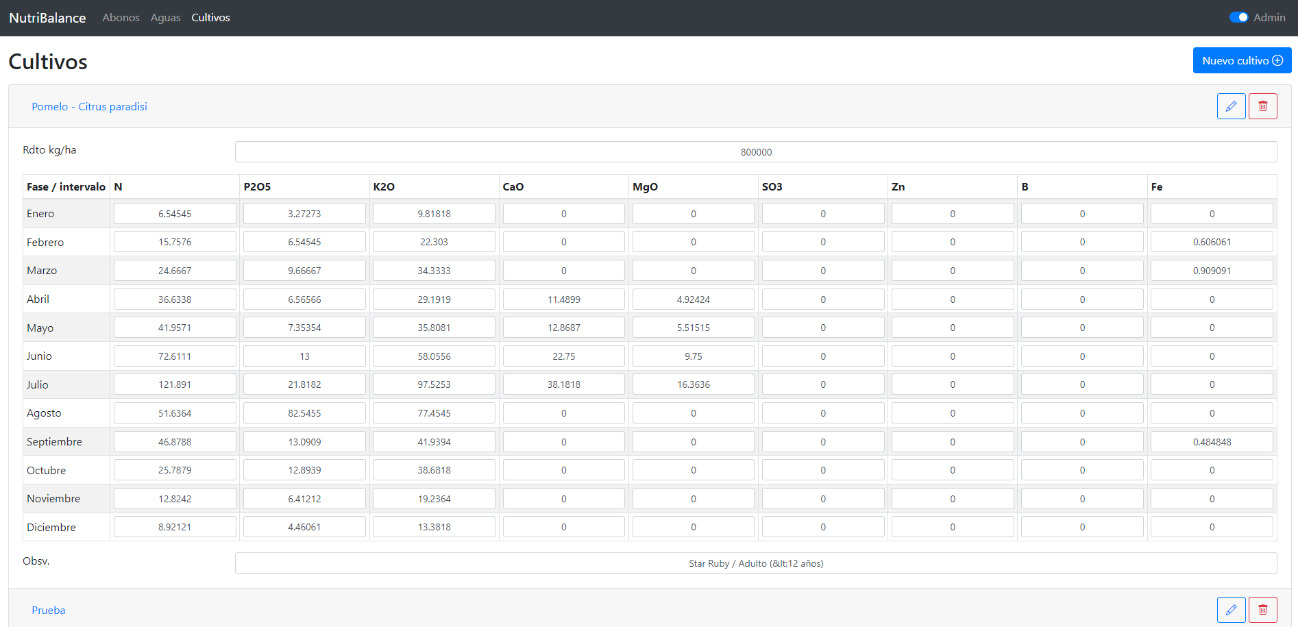

Supplement: Trial version link of NutriBalance [file DataSheet_1.zip › Data Sheet 2/Supplementary Figure S3.jpg]

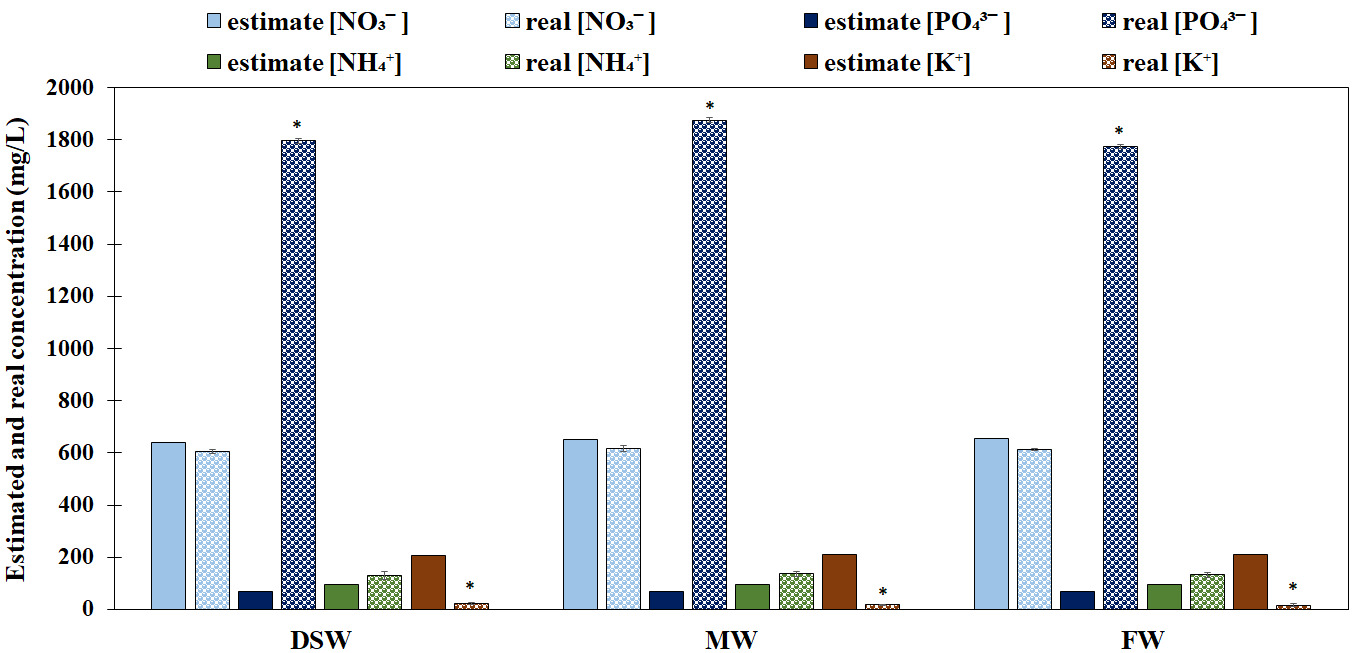

Supplement: Trial version link of NutriBalance [file DataSheet_1.zip › Data Sheet 2/Supplementary Figure S4.jpg]

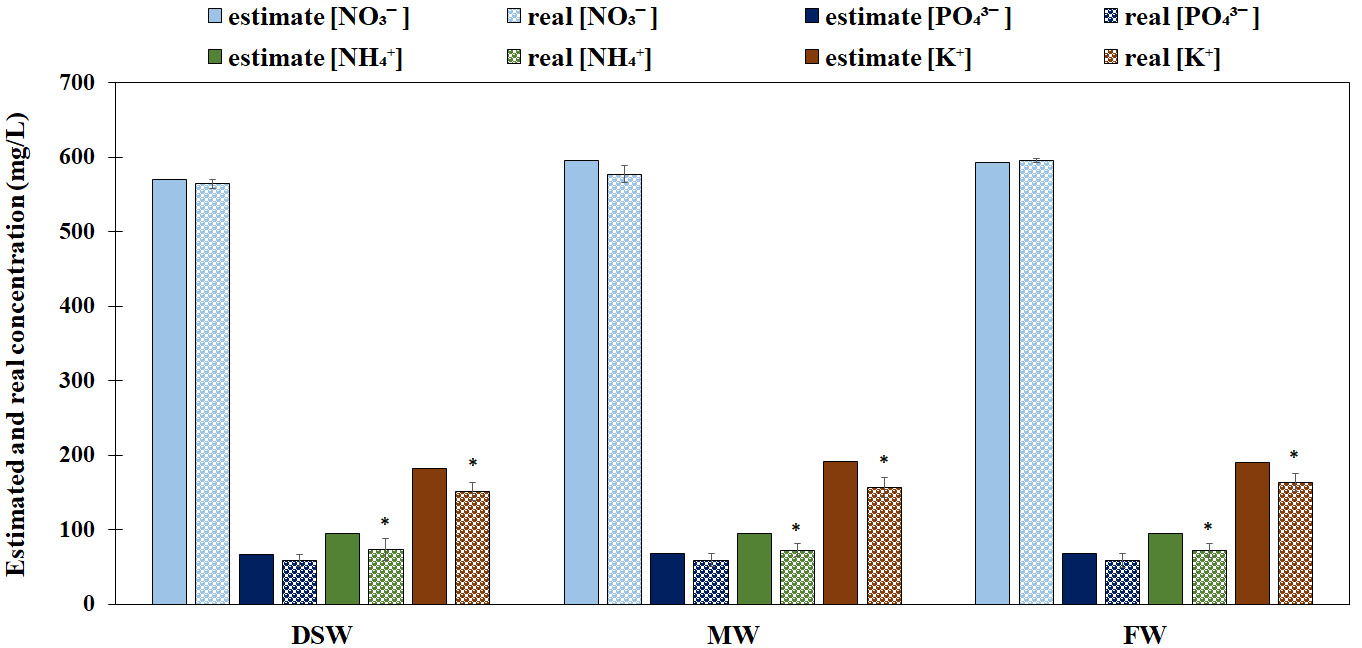

Supplement: Trial version link of NutriBalance [file DataSheet_1.zip › Data Sheet 2/Supplementary Figure S5.jpg]

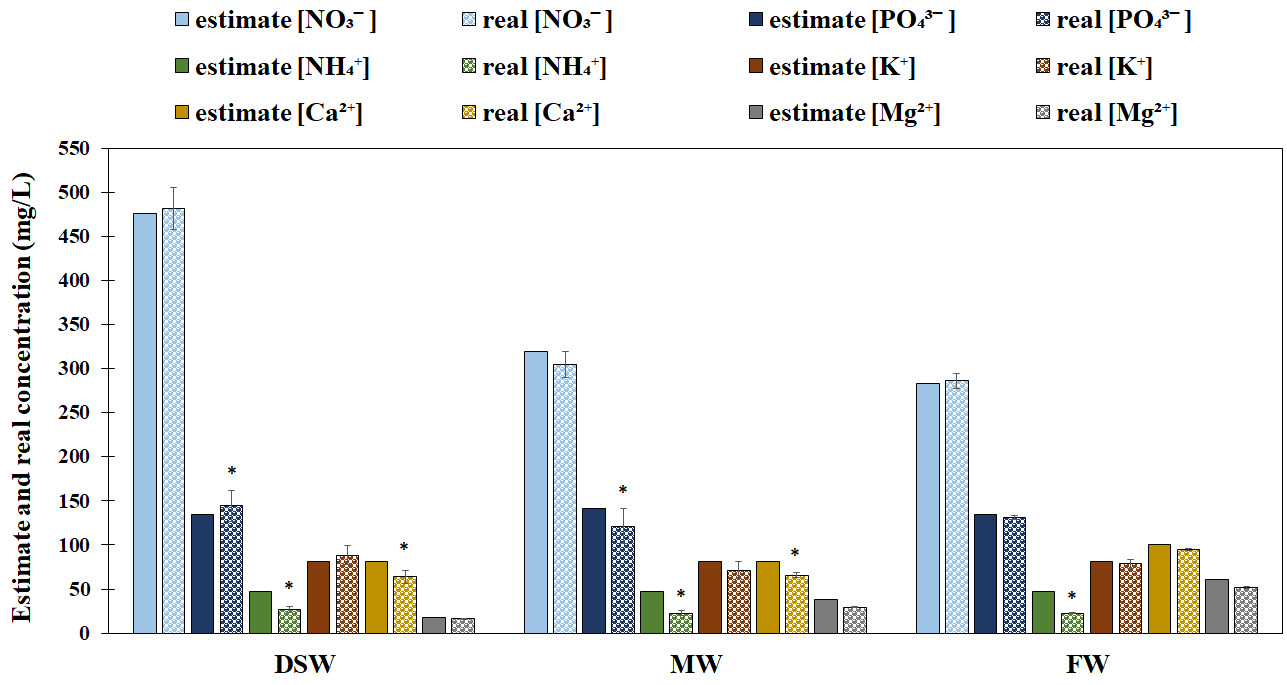

Supplement: Trial version link of NutriBalance [file DataSheet_1.zip › Data Sheet 2/Supplementary Figure S6.jpg]

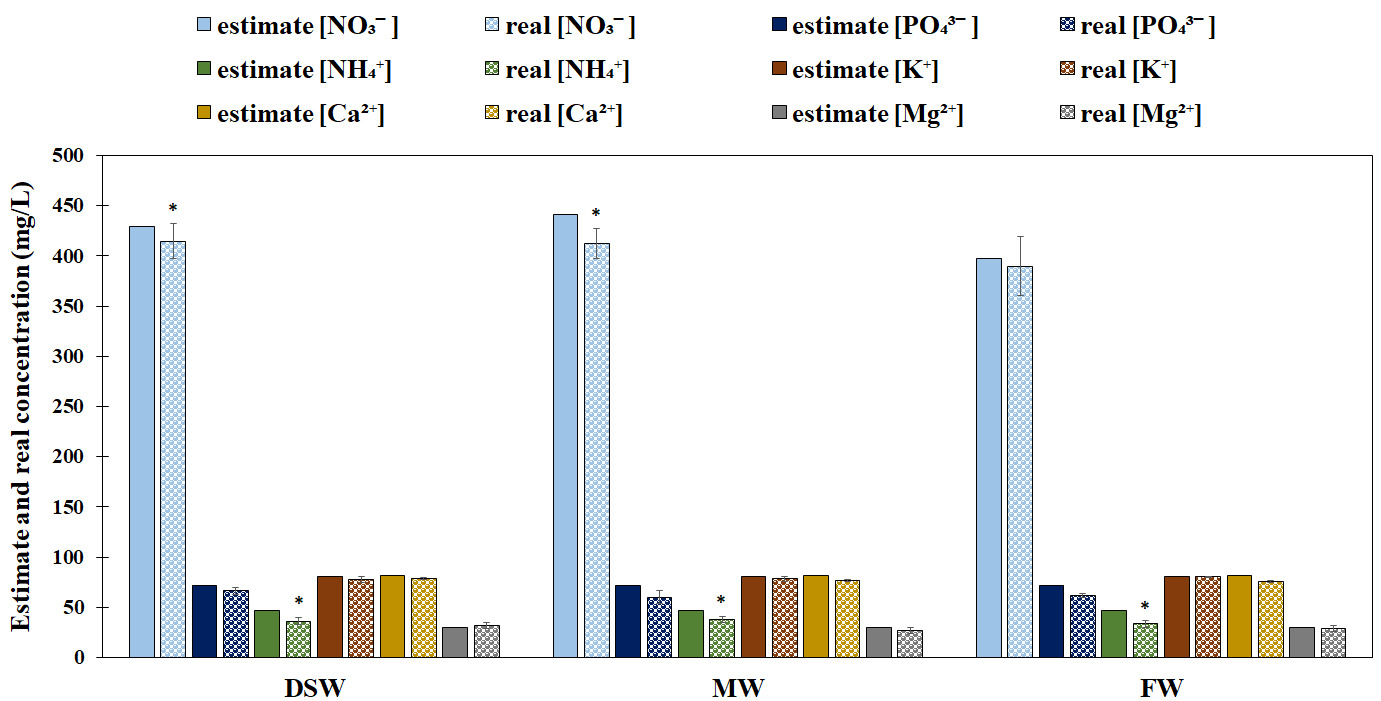

Supplement: Trial version link of NutriBalance [file DataSheet_1.zip › Data Sheet 2/Supplementary Figure S7.jpg]
